# Farmers’ risk preferences and rice production: Experimental and panel data evidence from Uganda

**DOI:** 10.1371/journal.pone.0219202

**Published:** 2019-07-08

**Authors:** Yoko Kijima

**Affiliations:** National Graduate Institute for Policy Studies (GRIPS), Tokyo, Japan; International Food Policy Research Institute, UNITED STATES

## Abstract

Although rice has been a prominent cash crop in areas with access to lowland in Uganda, the adoption of rice and area expansion have stagnated despite the Government of Uganda’s 2009 National Rice Development Policy and its commitment to doubling rice production over 10 years. Using panel data collected in 2010 and 2017 as well as risk preference data elicited via lab-in-the-field experiments conducted in rural Uganda, we find that farmers with higher loss aversion are less likely to grow rice and expand their rice cultivation areas. This study affirms that risk preferences play a critical role in agricultural production decisions.

## Introduction

Many studies have examined why apparently profitable technology is not adopted. While theoretical studies have attributed low technology adoption in developing countries to many factors, high risk-aversion is one of them [1,2]. Several empirical studies test their theoretical predictions using household assets and educational attainment as proxies for risk aversion since they are key determinants of it [3,4]. Other studies have adopted survey-based methods whereby hypothetical choice questions with different degrees of risk are used to measure risk aversion [5]. Since the survey-based method is not incentive-compatible, self-reported personal attitudes and traits may not capture actual risk preference levels [6]. Thus, studies have attempted to measure risk aversion using experimental methods [7].

Several experimental methods have been developed to observe choices that reflect each individual’s risk preference [8]. Experiments in which many of the experimental factors are controlled by the experimenter can ensure that the elicited risk measure is influenced only by risk preferences. The experimental evidence on risk preferences is growing, but studies that examine whether risk attitudes affect agricultural technology adoption decisions using elicited risk preferences are scant.

**Fig 1:**
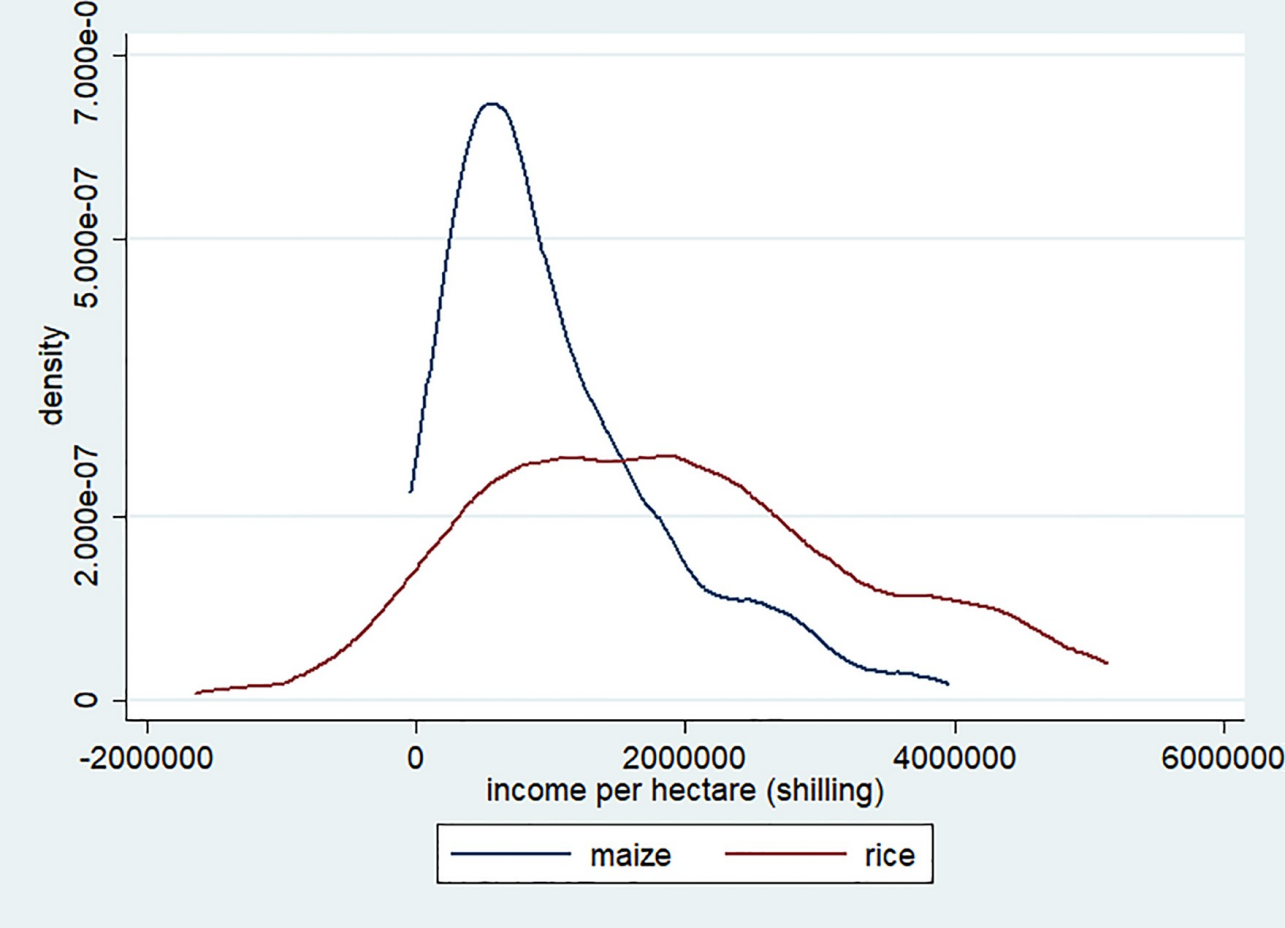
Rice and maize income. Household-level rice and maize income in 2009/10 calculated by deducting paid-out costs from revenue (total production multiplied by median price at each village). Income measured at 2010 price level, and density estimated by using kdensity command in Stata software.

This study examines whether risk preferences affect rice adoption in Uganda using panel data collected in 2010 and 2017 as well as risk preferences elicited from lab-in-the-field experiments. Although rice has been a prominent cash crop in areas with access to lowland in Uganda [9], rice adoption and area expansion have stagnated. This is disappointing given that the Government of Uganda (GoU) released the National Rice Development Policy (NRDP) in 2009 and made a commitment to doubling rice production over 10 years. Many farmers hire labor for rice production, unlike for other major staple crops such as maize and cassava, since it needs to be transplanted and harvested within a few days. As indicated in Fig 1, the likelihood of earning negative income in normal year is much higher for rice than for maize. The standard deviation of the income per hectare from rice is much larger than that from maize. The stagnant diffusion of rice in terms of the number of rice growers and the area under rice cultivation can be explained with reference to farmers’ risk preferences, especially their loss aversion. Thus, this study tests the hypothesis that farmers’ loss aversion plays an important role in the adoption of rice cultivation.

Studies that elicit risk preferences through experiments find that higher risk aversion is associated with a lower adoption of new technologies with higher subjective risk such as Bt cotton in China [10] and chemical fertilizer in Uganda [11]. However, these studies use cross-sectional estimation method and do not control for household unobserved heterogeneity such as farming ability and soil fertility, which can result in biased estimates. To our knowledge, ours is the first study to use panel estimation methods to analyze the role of risk preferences in agricultural technology adoption combined with elicited risk and loss aversion from a lab-in-the-field experiment. By applying a correlated random effect framework, we find that those who are more loss averse are less likely to adopt rice or to expand their cultivation area. Although our main variable of interest is rice adoption which is not technology adoption but crop choice, we also examine technology adoption such as better farming practices and chemical fertilizer for growing rice. We find no evidence that risk preferences decrease adoption rates of better farming practices and chemical fertilizer once we limit the sample to rice growers.

This study also examines whether risk preferences affect households’ diversification strategies for both rice cultivation and that of other crops. Households attempt to reduce risk by diversifying their income sources [12] as well as by allocating land to different crops [13] and labor to non-agricultural activities [14–16]. The amount of land to allocate to drought-tolerant crops is determined according to the household’s risk preferences. Accordingly, we find that more risk-averse households tend to allocate more land to leguminous crops. Regarding the diversification of income sources, we find no significant evidence that households displaying higher risk and loss aversion tend to diversify income to off-farm activities other than crop production.

The rest of this paper is organized as follows. Section 2 describes the study’s data, experimental design, and econometric framework. Section 3 provides the experimental results and estimation results. The final section presents conclusions.

## Methods

### Data

In Uganda, rice is a staple food, though it is a relatively minor source of calorie consumption [17]. Rapid population growth and urbanization have been increasing rice consumption levels, resulting in the annual importation of 60,000 metric ton of rice [18]. Since increasing rice production can be used to save foreign reserves by reducing the amount of imported rice as well as to improve food security and reduce rural poverty, the GoU formulated the NRDP in 2009 and committed to doubling rice production over 10 years by joining the Coalition for African Rice Development (CARD[19]).

**Fig 2:**
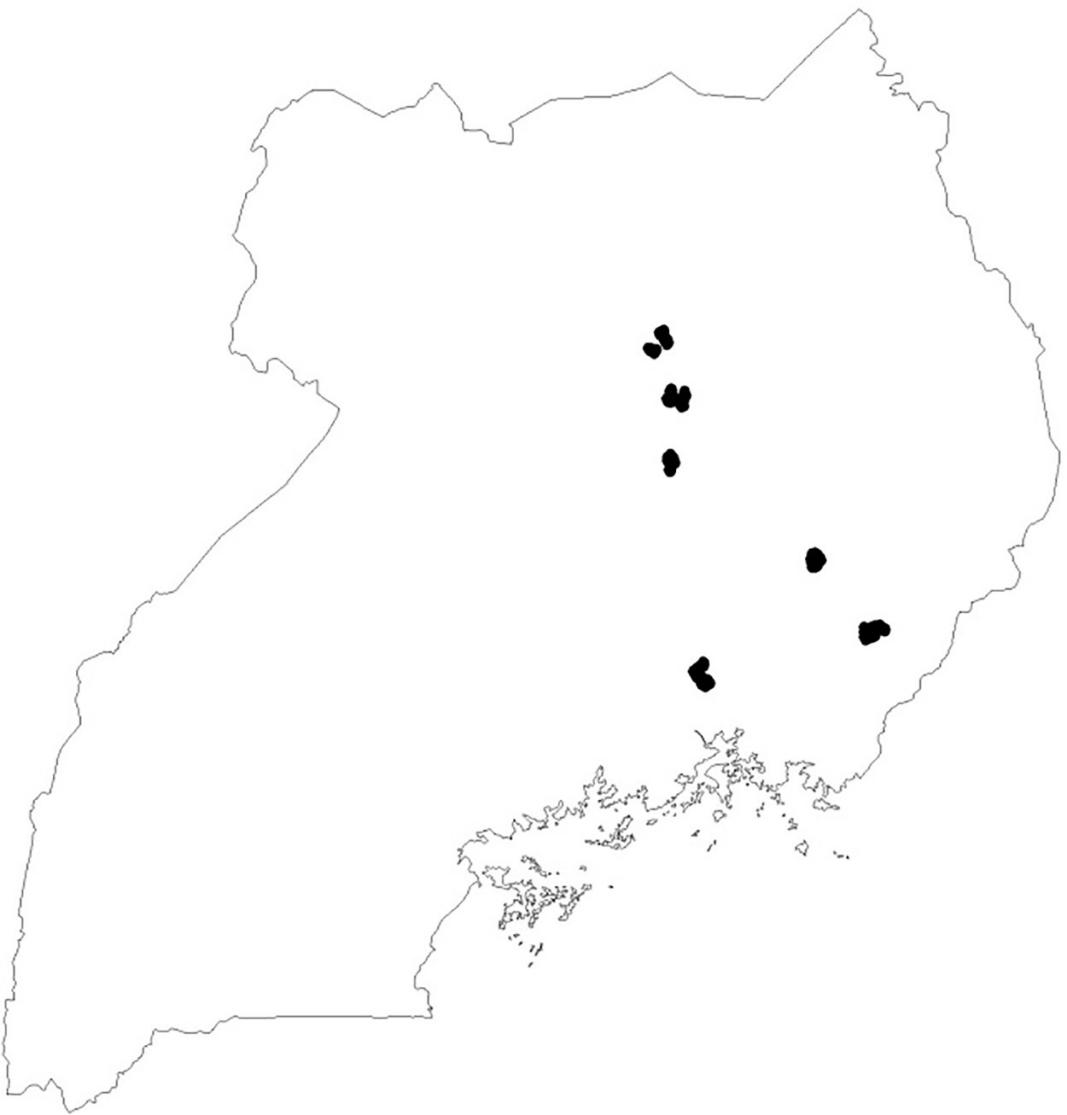
Sample communities. Black dots are locations of sample communities within the international boundary of Uganda. Source: Authors created this figure by using Arc GIS.

Household surveys were conducted in 2010 and 2017 to monitor the progress of rice production in Uganda amid its rainfed lowland ecosystem by constructing panel data in major lowland rice production areas. Structured questionnaire and procedures and scripts for field experiments were reviewed and approved by the research ethics committee at the JICA Research Institute. The sample districts were purposively selected based on the availability of the wetland usable for rice production in eastern and northern Uganda. The average rice cultivation experience and agro-ecological conditions were also used as criteria for selecting the sample districts, employed to capture a wide variety of rainfed lowlands and various levels of rice cultivation skill. Five districts out of 28 eastern and two northern districts were chosen (see Fig 2). The geographic coordinates for these districts are (00°56N, 33°57E), (01°30N, 33°57E) (01°55N, 33°06E), (02°20N, 33°06E), and (01°55N, 33°10E). Butaleja and Lira districts have large-scale irrigation schemes, and farmers in these districts have longer-term rice production experience than farmers in the other districts. Households in Lira and Dokolo districts also have larger landholdings, on average, than households in other districts.

We collected the information about main rice producing area from the District Agricultural Officer in each district. Two sub-counties locally well-known as rice producing areas were selected from each district. In the 10 selected sub-counties, the names of all Local Council 1s (LC1s, the lowest administrative unit in Uganda, consisting of three to five villages) in each sub-county were listed. From the list, 60 LC1s were randomly selected. In each LC1, 10 households were randomly selected using the household lists obtained from the LC1chairpersons. A total of 600 households were interviewed in January-February 2010. Before interviewing, enumerators asked sampled households if they agreed to be interviewed verbally and a few refused to be interviewed. In the follow-up survey conducted in February 2017, 61 households were not available for interview, mostly because they had moved far away or to unknown places. The panel data comprise 539 households. Since the attrition rate is about 10%, attrition weight will be used for all the analyses to address possible biases due to non-random attrition. A community-level survey was also conducted in each LC1.

The household questionnaire posed a wide range of questions to capture data on all of the farm and non-farm activities engaged in over the previous 12 months, as well as on household demography, consumption expenditures, and asset ownership (e.g., land, livestock, farm equipment, other household items). For farm activities, During the 2017 survey, all the sample households were invited after the interview to participate in incentivized lab-in-the-field experiments. We invited one person from each household who was either the household head or main decision-maker concerning crop production. When the household head was too old to understand the experimental design or engaged mainly in non-farm work, the survey team invited the non-household head who made the major decisions about farm work.

### Experimental design

In the experiments, participants chose from a price list. Risk preference elicitations were administered in a group setting in which the experimenter read instructions in front of the entire group. Each group had 10 subjects. Choices were made individually and privately. The subjects were paid at the end of the experiment, which provided no incentive to answer dishonestly [20]. Our multiple price list experiment was developed based on that in [8], which is relatively simple and easy to understand even by uneducated farmers in developing countries [21]. The participants chose one item from paired lottery options. As shown in S1 File, they were asked to pick either option A (safe option) or option B (risky option). The experiment was kept as simple as possible to prevent confusion over the concept of probability. This list had been tested before in Uganda [22] and was also pretested before this survey.

Two risk preference experiments were conducted: one involving payoff gains and the other involving both payoff gains and losses. One advantage of the experiments is that they were incentivized and involved real losses, just like real-life investments. The experimental procedure used in this study is comparable to previous experiments on risk-elicitation tasks. Table 1 indicates the different payoff options in the risk preference experiments. Each experiment comprised eight rows. The first question was a test designed to judge whether the respondent understood the instructions [23]. In Experiment1, the payoff associated with option A remained unchanged, while the payoff for option B increased. In Experiment 2, the payoff associated with option A declined systematically throughout the tasks, while the payoff for option B remained unchanged or increased. By assuming constant relative risk aversion, the respondents’ risk aversion is then directly related to the line at which they switched from preferring option A to preferring option B. The expected values of the two options were not shown to the respondents during the experiments.

**Table 1:** Payoff matrix for the risk preference experiments.

Panel A. Experiment 1 (Gains)										
Row	Column A		Column B				EV^A^ -EV^B^	CRRA interval if switches to B under EUT	Mid Point	Number of subjects
	p	M	p	M_H_	1-p	M_L_				
1–1	1	4000	0.25	4000	0.75	2000	1500	n/a	n/a	-
1–2	1	4000	0.25	7000	0.75	2000	750	-∞ < σ ≤ -1.15	-1.15	43
1–3	1	4000	0.25	10000	0.75	2000	0	-1.15 < σ ≤ 0.00	-0.58	72
1–4	1	4000	0.25	13000	0.75	2000	-750	0.00 < σ ≤ 0.41	0.21	37
1–5	1	4000	0.25	16000	0.75	2000	-1500	0.41 < σ ≤ 0.62	0.52	42
1–6	1	4000	0.25	16000	0.75	2000	-2250	0.62 < σ ≤ 1.60	1.11	88
1–7	1	4000	0.25	16000	0.75	2000	-2625	1.60 < σ ≤ 3.04	2.32	71
1–8	1	4000	0.25	16000	0.75	2000	-3000	3.04 < σ ≤ ∞	3.04	168
Panel B: Experiment 2 (Losses)										
Row	Column A				Column B				EV^A^ -EV^B^	Number of subjects
	p	M_H_	1-p	M_L_	p	M_H_	1-p	M_L_		
1–1	0.5	6000	0.5	-500	0.5	6000	0.5	-4000	1750	-
1–2	0.5	4000	0.5	-500	0.5	6000	0.5	-4000	750	4
1–3	0.5	1000	0.5	-500	0.5	6000	0.5	-4000	-750	89
1–4	0.5	500	0.5	-500	0.5	6000	0.5	-4000	-1000	51
1–5	0.5	500	0.5	-500	0.5	6000	0.5	-3000	-1500	63
1–6	0.5	500	0.5	-1000	0.5	6000	0.5	-3000	-1750	41
1–7	0.5	500	0.5	-1000	0.5	6000	0.5	-2000	-2250	83
1–8	0.5	500	0.5	-1000	0.5	6000	0.5	-1000	-2750	172

Notes: Panel shows all the payoffs (M) and the attached probabilities (p, 1-p) for choices A and B in the risk preference experiment 1 (Panel A) and experiment 2 (Panel B). EV^A^—EV^B^ is the difference in the expected value between lottery A and lottery B. The range of sigma σ is calculated by equating the expected utilities from lottery A and lottery B assuming constant relative risk aversion (CRRA) utility function.

A risk-seeking participant would choose the lottery in the second task, while the most risk-averse subject would choose option A in the eighth task. Because a risk-neutral participant should theoretically switch from option A to option B when the expected value of both is approximately equal, the participant would select option A for the third task. The respondents’ loss aversion is elicited from Experiment 2, and the risk aversion coefficient is elicited from Experiment 1. A detailed explanation is provided in S1 File. We do not examine ambiguity aversion, unlike the previous study [24], who found that ambiguity aversion is more important than risk aversion for the early adoption of genetically modified corn in the US, which decreases the ambiguity of pest damage. Since our sample households reside in communities characterized by rice cultivation, the risk of growing rice and its distribution are known factors, which makes ambiguity aversion is less relevant than risk aversion.

### Empirical model

To assess whether risk preferences affect farmers’ crop choices, we estimate the following model:

$$y_{ijt}\;=\;\beta X_{ij}+\gamma Z_{ijt}+\mu_{i}+e_{ijt}$$

where y_ijt_ is the crop choice of household *i* in village *j* at time t. X_ij_ is a set of the respondents’ elicited risk preferences (risk and loss aversion), Z_ijt_ is a set of household characteristics, μ_i_ is household-level unobserved heterogeneity other than risk preferences, and e_ijt_ is an error term. β and γ are the parameters to be estimated. The main coefficient of interest is β. We use three measures for crop choice: a dummy variable reflecting whether the household has cultivated rice in the last 12 months, the area under rice cultivation, and the share of land under rice cultivation. We use both risk and loss aversion coefficients as the main explanatory variable, similar to the previous studies [10, 25]. The results remain qualitatively the same when we use the risk preference measures one at one time as alternative specifications, as shown in S6 Table.

Since not all the households cultivated rice, our outcome variables are likely to be nonlinear. For the estimates to be consistent in nonlinear panel models, the assumption of independence between the covariates and μ_i_ needs to be held when using fixed effect models. However, the assumption is often too strong to be held. Alternatively, a correlated random effects framework does not require this assumption and can control for time-constant unobserved heterogeneity (as can the fixed effects model) without having incidental parameters in nonlinear models [26]. Since the main explanatory variables—the risk preference measures—are time-invariant, a correlated random effects framework that can estimate the time-constant independent variables is preferable to a household fixed effect model.

Even after time-constant unobserved heterogeneity μ_i_ is controlled for by adopting a correlated random effects framework, one may argue that risk preference X_ij_ is correlated with time-varying unobservable e_ijt_. As shown in S2 Table, risk preferences were not affected by recent shocks. It is thus safe to assume independence between risk preference X_ij_ and time-varying unobservable e_ijt_ after controlling for μ_i_.

Rice has been a cash crop in our sample areas for more than 30 years. For some households, not growing rice (i.e., having an area under rice cultivation of zero) can be optimal. As functional forms, we adopt a probit model to determine whether to cultivate rice and a Tobit model for the area under rice cultivation and the share of area under rice cultivation out of the total cultivated area.

Since 10% of the sample households could not be resurveyed in 2017, we adopt the attrition correction procedure suggested by [27] and use attrition weights in all analyses. We first estimate a probit model to explain whether a household remains in the follow-up survey and obtain the predicted probability that a household remains in the panel data. The attrition weights are calculated as the inverse of the predicted probabilities in order to give greater weight to households with a lower probability of remaining in the sample but who were in fact re-surveyed. The results of the probit model are shown in S1 Table.

To analyze crop production diversification, we consider the area that households have used for maize, root, and leguminous crop cultivation in the previous 12 months. Since root crops such as cassava are less prone to drought, households with higher risk aversion may plant cassava in a large area.

## Results

### Experimental results

The last column of Table 1 shows the distribution of the rows in which subjects switched from option A to B. We see that a significant number of subjects (168) are extremely risk averse. Those who switched in either row 7 or 8 account for 46% of the total. Such extreme choices are also found in Experiment 2, where 51% switched from A to B in either row 7 or 8. This suggests that the participants chose slightly safer options when the game involved losses.

In Experiment 1, 18 participants chose all A or all B, which are not rational choices. In Experiment 2, an additional 18 participants selected irrational choices. Since they might not have understood the risk experiment fully, we do not use these cases in our analyses. Thus, the total number of households used in the analyses of risk and loss aversion became 521 and 503, respectively.

Table 2 summarizes the estimated risk aversion and loss aversion parameters as well as the participants’ basic characteristics. About 70% of the participants were household heads, 66% were male, and they had completed 5.9 years of schooling. There were no differences in age or education levels between those who had grown rice and those who had never done so.

**Table 2:** Risk and loss aversion.

	Combined Mean	HH who have grown rice	HH who have never grown rice	t-stats
	(1)	(2)	(3)	(4)
Risk aversion	1.366	1.388	1.186	-0.958
	(1.497)	(1.497)	(1.496)	
Loss aversion	2.259	2.208	2.657	2.082
	(1.538)	(1.532)	(1.536)	
Number of observations	521	475	46	
Subject’s characteristics	46.354	46.877	46.293	0.287
Age	(14.511)	(15.254)	(14.436)	
Schooling	5.876	5.789	5.886	-0.193
	(3.555)	(4.118)	(3.488)	
Gender (Male = 1)	0.657	0.667	0.657	0.166
	(0.475)	(0.476)	(0.476)	
Head	0.688	0.684	0.689	-0.071
	(0.464)	(0.469)	(0.463)	

Note: Numbers in parentheses are standard deviations. The last column shows the t-statistics to test if means of column (2) and (3) are same or not.

The overall mean risk aversion (1.37) is comparable to the estimate of 1.32 for rural Uganda [28]. The mean loss aversion parameter (2.26) is lower than the estimates for rural Uganda (3.22) [22]. The notion of loss aversion is that the disutility of losses weighs more heavily than does the utility of comparable gains [29, 30]. The results suggest that, on average, the reduction in utility due to losing 1 USD has a magnitude equal to an increase in utility due to gaining 2.26 USD. As shown in columns 2 and 3, no differences in risk aversion are observed between rice growing households and non-growing households, while rice growers tend to be less loss averse than non-growers.

Following [8], we examine the correlates of risk preferences via ordinary least square (OLS). S2 Table presents the estimated results. None of the individual or household characteristics except marital status is significantly associated with risk preferences. Those who are married are more likely to be less loss averse. Furthermore, shocks exposed in the last 12 months are not correlated with risk preferences, suggesting that risk preferences are stable to exogenous shocks [31].

### Descriptive statistics

Table 3 presents the descriptive statistics for participant, household, and community characteristics. About 10% of the sample households have never grown rice. Households who have grown rice have more members than those who have never done so. There are no differences in education level, assets, land holdings, access to off-farm employment and non-labor income, or access to credit (measured by bank account holder and saving group member) between rice growers and non-growers. However, rice growers are better equipped in terms of access to agricultural training, phone ownership, and access to district towns. The bottom rows of Table 3 show whether the households experienced shocks such as drought, floods, land conflict, and sickness in the last 12 months. Half of the households experienced drought in the last 12 months. There are no differences in the share of households who were recently exposed to shocks between rice growers and non-rice growers. In the sample areas, certified lowland rice variety seeds are not sold by agro-dealers. Most of the farmers keep their own seeds out of their harvests or purchase them from fellow farmers. Although we asked the farmers what variety they cultivated in the survey, many were not sure.

**Table 3:** Descriptive statistics.

	Combined Mean	HH who never grown rice	HH who have grown rice	t-stats
Head’s age	51.087	51.667	51.019	0.341
	(13.559)	(14.361)	(13.476)	
Head Schooling	5.757	5.561	5.780	-0.433
	(3.601)	(4.148)	(3.534)	
Female Headed HH	0.096	0.158	0.089	1.662
	(0.296)	(0.368)	(0.285)	
Household Size	7.289	6.368	7.398	-2.579
	(2.866)	(2.717)	(2.866)	
Share of male adults (15–69)	0.277	0.285	0.276	0.388
	(0.159)	(0.212)	(0.162)	
Share of female adults (15–69)	0.255	0.298	0.249	2.424
	(0.143)	(0.174)	(0.138)	
Own Land Size (Acre)	5.395	5.171	5.422	-0.328
	(5.455)	(3.948)	(5.609)	
Assets Value (100,000 Uganda Shillings at 2010 price level)	6.629	8.741	6.432	1.331
	(11.261)	(16.897)	(10.588)	
Received non-labor income	0.866	0.842	0.869	-0.569
	(0.341)	(0.368)	(0.337)	
Has HH member engaged in off-farm employment	0.733	0.719	0.734	-0.244
	(0.443)	(0.453)	(0.442)	
Member of HH has phone	0.412	0.263	0.429	-2.421
	(0.493)	(0.444)	(0.496)	
Saving group member (ROSCA or VSLA)	0.518	0.544	0.515	0.418
	(0.500)	(0.503)	(0.500)	
Agricultural Training (last 5yrs)	0.430	0.281	0.448	-2.423
	(0.496)	(0.453)	(0.498)	
Farmer group member	0.234	0.298	0.226	1.216
	(0.424)	(0.462)	(0.419)	
Distance from home to district town (km)	21.038	23.632	20.731	2.127
	(9.769)	(11.023)	(9.576)	
Shocks experienced (last 12 months) Drought	0.503	0.474	0.506	-0.464
	(0.500)	(0.504)	(0.500)	
Shocks experienced (last 12 months) Floods	0.029	0.000	0.033	-1.396
	(0.169)	(0.000)	(0.179)	

Note: In parenthesis are standard deviations. t-statistics for testing means between those who have grown rice by January 2017 and those who have never grown rice.

Table 4 shows the rice cultivation results for 2009 and 2016. Although nearly 90% of the sample households have grown rice, as shown in Table 3, the proportion of households who cultivated rice in the previous 12 months decreased from 67% in 2009 to 58% in 2016. The area under rice cultivation and the share of the area under rice cultivation have not increased. Among rice growers, around 1 acre of land is allocated to rice, which accounts for 18% to 20% of the cultivated land (see Panel B). In the sample area, there is no evidence that the number of rice growing households or the area under rice cultivation expanded from 2009 to 2016.

**Table 4:** Rice cultivation and input use in 2009 and 2016.

	2009		2016	
**Panel A. All households (No. HH = 503)**				
Households who grew rice in the last 12 months	0.672	(0.470)	0.584	(0.493)
Area under rice (ha)	0.400	(0.597)	0.381	(0.545)
Share of area under rice	0.164	(0.177)	0.175	(0.209)
Share of land allocated to maize	0.231	(0.193)	0.239	(0.256)
Share of land allocated to roots crops	0.181	(0.165)	0.194	(0.231)
Share of land allocated to legumes	0.139	(0.128)	0.145	(0.195)
Area under maize (ha)	0.435	(0.621)	0.465	(0.635)
Area under legume crops (ha)	0.333	(0.505)	0.295	(0.510)
Area under roots crops (ha)	0.474	(0.634)	0.326	(0.442)
Labor hired in the last 12 months (any crop)	0.636	(0.481)	0.496	(0.500)
Labor hired for upland crop in the last 12 months	0.353	(0.478)	0.498	(0.500)
Chemical fertilizer applied (any crop)	0.030	(0.170)	0.169	(0.375)
Share of income from crop production and farm labor	0.706	(0.260)	0.625	(0.291)
Share of income from livestock activities	0.155	(0.276)	0.148	(0.187)
Share of income from non-farm sector	0.119	(0.244)	0.228	(0.299)
Share of non-labor income	0.020	(0.142)	0.000	(0.001)
**Panel B: Rice growers only (No. HH = 388)**				
Households who grew rice in the last 12 months	0.737	(0.441)	0.654	(0.476)
Area under rice (ha)	0.442	(0.616)	0.426	(0.560)
Share of area under rice	0.180	(0.179)	0.196	(0.212)
Adoption of cultivation practices				
Constructing bunds	0.536	(0.499)	0.546	(0.499)
Broadcasting	0.434	(0.496)	0.435	(0.497)
Transplanting in row	0.050	(0.218)	0.089	(0.285)
Rice yield (ton/ha)	2.112	(1.328)	1.494	(1.439)
Rice income (mill. shilling/ha)	1.694	(1.591)	1.576	(1.993)

Numbers in parentheses are standard deviations.

As a measure of better cultivation practices (see Panel B of Table 4), we use two measures to reflect whether households constructed bunds for better water control and transplanted in rows for proper plant density and easier weed management. More than half of the households have adopted a transplanting method, but less than 10% transplant in rows. About 40% of them use the broadcasting method, which can save time and labor but makes it difficult to control weeds without using herbicide. Rice productivity is measured in terms of rice production per hectare (yield) and rice income (revenue minus paid-out costs) per hectare. In 2016, many households could not harvest rice due to severe drought, lowering the average yield and income in 2016 relative to the 2009 figures.

As shown in Panel A of Table 4, households allocate more land to maize than to rice. Roots crops such as cassava and sweet potato and leguminous crops such as common beans and ground nuts are also commonly grown in the sample communities. Around 70% of household income was generated from crop production and wage earning from farm labor in 2009. In 2016, when severe drought affected many households, the share of crop income declined, and the share of non-farm income increased.

### Estimation results

Table 5 shows the estimation results regarding rice cultivation decisions generated by estimating correlated random effects model. In columns (1) and (2), the dependent variable is a dummy variable reflecting whether the households cultivated rice over the previous 12 months. The dependent variable in columns (3) and (4) is the share of the area under rice cultivation, while that in columns (5) and (6) is the area under rice cultivation in hectares, respectively. We show two specifications for each dependent variable. In the second specification (columns 2, 4, and 6), access to agricultural information (farmer group membership) and access to credit (ROSCA or VSLA) are added as explanatory variables.

**Table 5:** Rice cultivation (correlated random effect).

	Rice grown in last 12 months Probit model (dy/dx)		Share of area under rice in last 12 months Tobit model (dy/dx)		Area under rice (ha) in last 12 months Tobit model (dy/dx)	
	(1)	(2)	(3)	(4)	(5)	(6)
Risk aversion	-0.0323	-0.0282	-0.0112	-0.0110	-0.0327*	-0.0306*
	(0.0416)	(0.0417)	(0.00860)	(0.00856)	(0.0181)	(0.0180)
Loss aversion	-0.124***	-0.119***	-0.0271***	-0.0270***	-0.0760***	-0.0746***
	(0.0392)	(0.0392)	(0.00833)	(0.00830)	(0.0176)	(0.0175)
Head Age	-0.016***	-0.0164***	-0.0026***	-0.0027***	-0.0064***	-0.0066***
	(0.00445)	(0.00445)	(0.00096)	(0.000953)	(0.00204)	(0.00203)
Head Schooling	-0.0219	-0.0250	-0.00721*	-0.0075**	-0.00514	-0.00695
	(0.0179)	(0.0180)	(0.00373)	(0.00374)	(0.00784)	(0.00785)
Female Head	0.173	0.157	0.0573	0.0565	-0.0264	-0.0310
	(0.342)	(0.341)	(0.0742)	(0.0739)	(0.155)	(0.154)
Household Size	0.0313	0.0265	-0.0001	0.00085	0.0312***	0.0318***
	(0.0281)	(0.0285)	(0.00553)	(0.00558)	(0.0115)	(0.0116)
Share of males (15–69)	0.450	0.449	0.0387	0.0343	0.134	0.105
	(0.493)	(0.493)	(0.101)	(0.101)	(0.213)	(0.212)
Share of females (15–69)	0.0160	-0.0532	-0.210*	-0.199	-0.141	-0.128
	(0.586)	(0.591)	(0.125)	(0.126)	(0.262)	(0.262)
log(Landholding in ha +0.01)	0.110*	0.111*	-0.0199*	-0.0193*	0.0672***	0.0689***
	(0.0596)	(0.0597)	(0.0116)	(0.0115)	(0.0240)	(0.0239)
Value of assets (log)	0.0904	0.0950	0.0111	0.0105	0.0439	0.0433
	(0.0599)	(0.0602)	(0.0124)	(0.0124)	(0.0273)	(0.0272)
Non labor income	-0.0351	-0.0691	-0.0193	-0.0120	-0.0478	-0.0357
	(0.156)	(0.160)	(0.0329)	(0.0333)	(0.0679)	(0.0685)
Off farm employment	-0.0651	-0.0699	0.0149	0.0170	0.0324	0.0341
	(0.166)	(0.166)	(0.0343)	(0.0342)	(0.0708)	(0.0705)
No mobile phone	0.0975	0.0920	-0.0146	-0.0128	0.0301	0.0341
	(0.166)	(0.166)	(0.0344)	(0.0343)	(0.0711)	(0.0708)
Farmer group member		0.0854		-0.0481		-0.145**
		(0.179)		(0.0361)		(0.0735)
Saving group member		0.182		-0.0194		0.0380
(ROSCA or VSLA)		(0.178)		(0.0364)		(0.0747)
Year fixed effect	Yes	Yes	Yes	Yes	Yes	Yes
LC1 fixed effects	Yes	Yes	Yes	Yes	Yes	Yes
Observations	1006	1006	1006	1006	1006	1006

Note: Numbers in parentheses are z-statistics. Numbers shown are marginal effects.

***, **, and * indicate significance at 1, 5, and 10%, respectively. Means of all time varying factors (except risk and loss aversion) are controlled for. Attrition weights are used.

In both specifications, we find that higher loss aversion decreases the probability of rice cultivation. By contrast, there is no evidence that risk aversion influences the decision on whether to grow rice. The results estimated separately in 2009 and 2016 show similar results (see S3 Table). As an alternative specification, we run a conditional probit model to test whether risk preferences affect the rice-cultivation decision differently for rice growers and non-growers. Specifically, we test whether higher loss aversion decreases the likelihood of rice cultivation in 2016 given the adoption of rice in 2009. Similarly, we examine whether lower loss aversion increases the likelihood of rice cultivation in 2016 given the non-cultivation of rice in 2009.

S4 Table shows that among rice growing households in 2009, the higher loss aversion increases the probability of disadopting rice cultivation (see columns 1 and 2) while there is no evidence that among non-rice growing households in 2009, lower loss aversion increases the adoption rate of rice (see columns 3 and 4). These results suggest that risk preferences affect the adoption of rice differently between rice growers and non-growers.

We also look at the differential impact of risk and loss aversion on adopting rice by household’s wealth status. As shown in S2 Table, the wealth measured by the value of assets is negatively correlated with risk and loss aversion though the coefficients are not statistically significant. It may be the case that risk preferences affect household’s decision on rice cultivation differently by wealth status since poorer households tend to be more vulnerable to negative shocks [12]. Sample households are divided into two based on the value of assets. Average risk and loss aversion coefficients for poorer and richer households are not statistically different. We estimate same model separately for poorer households with asset values lower than the median and richer households with asset values higher than the median. As shown in S5 Table, the higher loss aversion decreases the probability of growing rice among the poorer households. Among richer households, however, we do not find such negative effect of loss aversion on rice cultivation decision. Thus, this finding suggests that households are less likely to grow rice if households with higher loss aversion face credit constraints.

The estimated results on the share of the area under rice cultivation out of the total cultivation area are presented in columns (3) and (4) of Table 5. We find that higher loss aversion decreases the share of the area under rice cultivation. As shown in the last 2 columns, higher loss aversion decreases the size of the land allocated to rice. Unlike in the results on the share of the area under rice cultivation, household size and landholding can be constraining factors on the expansion of the rice cultivation area.

Even when we control for risk aversion and loss aversion one at a time (S5 Table), loss aversion significantly affects the rice cultivation decision, but we find no evidence that risk aversion decreases rice adoption. To examine the heterogeneous effect of risk preference on rice cultivation, we estimate the model with an interaction term between risk preference and irrigation access. The coefficients of the interaction terms are not significant (see S6 Table).

Do risk preferences affect the adoption of better cultivation practices and increased productivity among rice growers? To answer this question, we adopt the same model as above but with different dependent variables. We use dummy variables indicating whether a household constructed bunds, broadcasted seeds, and transplanted in rows, if it applied chemical fertilizer, and the magnitude of the rice harvest and income per hectare.

Table 6 indicates the estimation results by limiting the sample to rice growers. Column 1 shows that those who have higher risk aversion tend to adopt better cultivation practices (e.g., constructing bunds). Those who have higher loss aversion tend to adopt transplanting in row method (Column 3). There is, however, no evidence that risk preferences affect the adoption of chemical fertilizer and broadcasting method. Rather, rice cultivation experience increases adoption of such cultivation practices. As shown in columns 5 and 6, we find no evidence that risk preferences affect rice productivity. A strong inverse relationship is observed between plot size and rice productivity. In the previous tables, we find that those who have higher loss aversion are less likely to grow rice on average.

**Table 6:** Adoption of rice cultivation practices and rice productivity.

	Bunds	Broadcast	Transplant in row	Chemical fertilizer use	Yield (ton) per hectare	Rice income (million shilling) per hectare
	(1)	(2)	(3)	(4)	(5)	(6)
Risk aversion	0.122*	-0.123	0.0567	0.0137	-0.0299	-0.0192
	(0.0671)	(0.155)	(0.0959)	(0.0106)	(0.0435)	(0.0574)
Loss aversion	0.0191	-0.106	0.218**	0.00837	0.0613	0.0275
	(0.0651)	(0.158)	(0.0983)	(0.0106)	(0.0466)	(0.0615)
Area under rice (ha)	0.359*	0.0148	0.367	-0.00476	-0.459***	-0.352*
	(0.201)	(0.260)	(0.308)	(0.0336)	(0.147)	(0.193)
Rice experience (yrs)	0.0489***	-0.148***	0.0408**	0.00586***	0.0133	0.0143
	(0.0127)	(0.0343)	(0.0176)	(0.00183)	(0.00846)	(0.0111)
Year fixed effect	Yes	Yes	Yes	Yes	Yes	Yes
LC1 fixed effects	Yes	Yes	Yes	Yes	Yes	Yes
Observations	460	460	460	460	460	460

Note: Numbers in parentheses are standard errors. Numbers shown are marginal effects.

***, **, and * indicate significance at 1, 5, and 10%, respectively. Attrition weights are used. Other controls are household head’s age, education, female headed household, household size, share of male adult members, share of female adult members, land owned, value of assets, non-labor income, off-farm employment, not owning mobile phone. Column 1–4 are estimated by correlated random effect probit model while column 5 and 6 are by correlated random effect tobit model. Sample: rice growers in 2009 and 2016.

To further explore how risk preferences affect land use (crop choice), we examine the share of upland cultivated area and the size of the land allocated to crops other than rice. As shown in Table 7, risk preferences do not affect the cultivation of maize and legume crops (e.g., beans, ground nuts). However, root crops (e.g., cassava, sweet potatoes) are positively associated with loss aversion. Higher loss aversion increases the size of the land allocated to root crops. Since root crops are less prone to drought and constitute a main food crop in Uganda, this result suggests that those who are more loss averse tend to be subsistence farmers.

**Table 7:** Crop choice other than rice (share of area allocated to other crops).

	Share of cultivated upland area			Area (ha)		
	maize	roots	legume	maize	roots	legume
	(1)	(2)	(3)	(4)	(5)	(6)
Risk aversion	-0.00144	0.00341	0.00702	-0.00685	0.00413	0.00768
	(0.00492)	(0.00519)	(0.00397)	(0.0117)	(0.00960)	(0.00915)
Loss aversion	0.00166	0.00505	-0.00264	-0.00526	0.0174*	-0.00256
	(0.00475)	(0.00495)	(0.00379)	(0.0113)	(0.00915)	(0.00872)
Year fixed effect	Yes	Yes	Yes	Yes	Yes	Yes
LC1 Fixed Effects	Yes	Yes	Yes	Yes	Yes	Yes
Mean in 2009 (s.d.)	0.222 (0.186)	0.293 (0.230)	0.166 (0.144)	0.460 (0.738)	0.487 (0.692)	0.345 (0.580)
Mean in 2016 (s.d.)	0.299 (0.294)	0.244 (0.281)	0.178 (0.217)	0.465 (0.635)	0.326 (0.442)	0.295 (0.510)
Observations	1006	1006	1006	1005	1005	1005

Note: Numbers in parentheses are standard errors. Estimated by correlated random effect tobit model. Numbers shown are marginal effects.

* indicate significance at 10% Means of all time varying factors (except risk and loss aversion and Muslim dummy) are controlled for. Attrition weights are used. Other controls are household head’s age, education, female headed household, household size, share of male adult members, share of female adult members, land owned, value of assets, non-labor income, off-farm employment, not owning mobile phone.

Finally, we assess how households make diversification decisions not only for crop production but also for other income sources. As explained earlier, we divide income into four sources: agriculture (income from crop production and farm labor wages), livestock, non-farm activities, and non-labor income. We estimate the same model to test if risk preferences affect income diversification or not. As shown in Table 8, no evidence is found that risk preferences increase the share of a specific income source.

**Table 8:** Share of income by source.

	Agriculture	Livestock	Non-farm	Non-labor income
	(1)	(2)	(3)	(4)
Risk aversion	-0.00401	0.00494	0.0203	-0.000897
	(0.00584)	(0.00631)	(0.0135)	(0.00312)
Loss aversion	-0.00670	-0.00303	0.0178	-0.00167
	(0.00559)	(0.00603)	(0.0125)	(0.00297)
Year fixed effect	Yes	Yes	Yes	Yes
LC1 fixed effects	Yes	Yes	Yes	Yes
Mean in 2009 (s.d.)	0.706 (0.260)	0.155 (0.276)	0.119 (0.244)	0.020 (0.142)
Mean in 2016 (s.d.)	0.625 (0.291)	0.148 (0.187)	0.228 (0.299)	0.000 (0.001)
Observations	1006	1006	1006	1006

Note: Numbers in parentheses are standard errors. Estimated by correlated random effect tobit model. Numbers shown are marginal effects. Means of all time varying factors (except risk and loss aversion and Muslim dummy) are controlled for. Attrition weights are used. Other controls are household head’s age, education, female headed household, household size, share of male adult members, share of female adult members, land owned, value of assets, non-labor income, off-farm employment, not owning mobile phone.

## Conclusions

This study examined whether risk-taking decisions are explained by risk preferences elicited by lab-in-the-field experiments in rural Uganda conducted in 2017, when many households were affected by severe drought. Under rainfed conditions in which agricultural production is subject to high risk, households’ risk attitudes are expected to influence crop choices because each crop is associated with a different risk level. In our sample communities, lowland rice cultivation is a profitable cash crop but has a higher risk of negative income during drought than other cash crops have because it requires households to hire labor to ensure timely harvesting and planting. It is thus hypothesized that the higher paid-out costs required for rice cultivation with an increased risk of zero harvest during drought conditions can prevent households characterized by higher risk and loss aversion from growing rice.

Using panel data collected in 2010 and 2017, we estimated the effect of risk preferences on crop and input choices via a correlated random effects model to consider unobserved time-invariant individual heterogeneity such as farming ability. Our results show that households with higher loss aversion are less likely to grow rice or expand rice cultivation. Among rice growers, however, risk preferences do not affect the decision on whether to adopt better cultivation practices or enhance rice productivity as measured by yield and income, respectively. Since households can reduce risk through diversification, we also examine whether households displaying higher risk and loss aversion allocate more land to crops with lower risks of negative income and engage in activities other than crop production. Our results show that households displaying higher loss aversion tend to allocate more land to roots crops, but we found no evidence of income diversification.

Given that higher loss aversion deters the expansion of rice cultivation, fostering such an expansion requires interventions designed to reduce risk and losses such as crop insurance, investments in irrigation facilities, and the development and diffusion of drought-tolerant rice varieties.

## Supporting information

S1 File Additional details on experiments. (PDF)

S1 Table Attrition. (PDF)

S2 Table Correlates of risk preferences. (PDF)

S3 Table Determinants of rice cultivation (cross section data). (PDF)

S4 Table Disadoption of rice in 2016 and adoption in 2016. (PDF)

S5 Table Rice cultivation by wealth category. (PDF)

S6 Table Determinants of area under rice (ha). (PDF)

S1 Data Dataset. (DTA)
