# Supplementary material for: Farmers’ risk preferences and rice production: Experimental and panel data evidence from Uganda
Source: PLoS One. 2019 Jul 8;14(7):e0219202. doi: 10.1371/journal.pone.0219202 (PMC6613747; doi:10.1371/journal.pone.0219202)
Supplement: S1 File — (PDF) [file pone.0219202.s001.pdf]

S1 File. Additional Details on Experiments

Although this study focuses on risk preference, we conducted 2 risk experiments and 2 time experiments as well as dictator and trust game experiments in one session. We randomly assigned LC1s to sessions beginning with risk experiment and time experiments. Dictator and trust game experiments were conducted after risk and time experiments. Before beginning the experiments, it was emphasized to the subjects that one of the risk and time experiments and one of the dictator and trust game experiments were to be played ex post for actual stakes; consequently, based on their respective choices in the actual experiments, their monetary payoff would be determined by the outcome of that lottery. This incentive was in addition to a show-up fee of Ushs 5,000, which was intended to cover the opportunity cost of participating in the experiments and to encourage participation. Ushs 5,000 is approximately 2 days' worth of wages for a typical male farm laborer by following Cardenas and Carpenter (2008)' argument that payment of 1 to 2 days' wages for a half-day session to subjects participating in economic experiments induces salience among them.

For the risk and time experiments, subjects were requested to choose column A or column B. All experiments were conducted with all participating subjects duly present to avoid any cross-talk effects that could bias the experimental outcome. Given that the level of education attainment of the subjects was low (5.9 years of schooling), we made a strong assumption that the subjects would not adequately understand the concept of probabilities used in the experimental design. Subjects who had trouble filling out the answer sheets individually during the experiments were carefully helped by the enumerators, who cautiously avoided giving specific instructions to the subjects on how to answer. However, such subjects were almost negligible. Whereas the

enumerators were conversant in the local dialect, they had no formal connections with the respective communities, which minimized any bias in responses to the subjects. In addition, while extensive training was offered to the enumerators to fully acquaint them with the research design and experimental procedures, they were ignorant about the experimental hypothesis.

After completing the risk and time experiments, one game was randomly selected to be played for real cash using a bingo machine. Each subject rolled the bingo machine with one of four balls numbered 1, 2, 3, and 4 for selecting one experimental result for actual payment. Balls 1, 2, 3, and 4 represent risk preference experiment 1, risk preference experiment 2, time preference experiment 1, and time preference experiment 2, respectively. Once the experiment to be played for real cash was decided based on the first ball to come out of the bingo machine, subjects were again asked to roll the bingo machine with eight balls numbered 1, 2, 3, 4, 5, 6, 7, and 8 to decide which row was to be played for real cash.

Measuring Risk Preferences

Risk Aversion

According to Holt and Laury (2002), the relative risk aversion parameter is often estimated to represent the degree of risk aversion. When a constant relative risk aversion utility function, $u(M) = \frac{M^{1-\sigma}}{1-\sigma}$ is assumed, where σ indicates the curvature of the utility function, and M is income. To compute the risk aversion parameter, σ, risk experiments are designed in such a way that they take the form of pair-wise choices or choices from a series of lotteries that include payoffs in each state i, M_i, with its probabilities p_i (Holt and Laury 2002). To compute the degree of risk aversion σ

reliably, we equate two lotteries that give the same level of expected utility $\sum_{i=1}^{n} p_i \frac{{M_i}^{1-\sigma}}{1-\sigma}$ to the individual and solve them simultaneously.

For risk preference experiment 1, which involved gains only, subjects were asked to choose between column A, which offered a sure payoff of Ushs 4,000 with 100% certainty, or column B, which offered two different payoffs with probabilities of either an extra 25% of payoffs in column 1 or 75% of payoffs in column 2. To ensure that subjects fully understood the notion of probabilities, four balls were used to demonstrate the probability concept. Figures A1 and A2 represent an excerpt of the risk preference experiments in which subjects made their respective payoff choices. The balls were numbered 1, 2, 3, and 4; they can be seen circled below A and B in each of the excerpts. Risk preference experiment 1 was elicited to measure the subject's degree of risk aversion, risk neutrality, or risk seeking, based on the point at which the subject switches from option A to B. For example, if a subject chose option A in rows 1-1, 1-2, 1-3, and 1-4, and chose option B in row 1-5, the subject's risk preference can be represented mathematically as follows:

$$\frac{4000^{1-\sigma}}{1-\sigma} \geq 0.25 \times \frac{13000^{1-\sigma}}{1-\sigma} + 0.75 \times \frac{2000^{1-\sigma}}{1-\sigma} \quad \text{.............. (1) from row 1-4}$$

$$\frac{4000^{1-\sigma}}{1-\sigma} \leq 0.25 \times \frac{16000^{1-\sigma}}{1-\sigma} + 0.75 \times \frac{2000^{1-\sigma}}{1-\sigma} \quad \text{.............. (2) from row 1-5}$$

Solving for equations (1) and (2) simultaneously, the interval of the risk aversion parameter is $0.41 < \sigma \leq 0.62$, and taking the mid-point, we obtain the risk aversion parameter as $\sigma = 0.52$.

In terms of real payment to the subjects after the experiments, for the sake of illustration, suppose that risk preference experiment 1, which involved gains only, was chosen to be played for real cash, implying that out of the four balls that were placed in the bingo machine, ball 1 came out of the bingo machine first. Then, when eight balls

were inserted in the bingo machine to determine which row would be played for real, and ball 5 came out, row 1-5 would be played for real. Subjects who had chosen option A in row 1-5 would be paid Ushs 4,000 with 100% certainty. For subjects who had chosen option B, four balls would be inserted in the bingo machine, and they would roll it again. If ball 1 came out first, the subjects would earn Ushs 16,000, while if balls 2, 3, or 4 came out first, the subjects would earn Ushs 2,000. Table 1 (Panel A) shows the payoff matrix for risk preference experiment 1. Subjects who chose A throughout are considered very risk averse, and therefore, are assigned $\sigma = 3.04$. Subjects who chose all B are considered very risk seeking and therefore, are assigned $\sigma = -1.15$.

Loss Aversion

Kahneman and Tversky (1979) in their seminal paper developed an alternative model from the expected utility theory, known as prospect theory. They argued that people knowingly underweight outcomes that are merely probable compared to outcomes that are certain. The loss aversion concept originates from this alternative model of prospect theory. The loss aversion concept has been described as the tendency of the prospect of losses to loom much larger than the prospects of gains of the same magnitude. This can be represented as *u(M) < -u(-M).* In economics experiments, just like risk aversion, loss aversion is elicited using lottery games. However, this involves negative payoffs as part of the choices (see Figure A2). The risk aversion parameter σ of each subject elicited in experiment 1 is utilized to estimate the level of loss aversion. Taking into consideration the mean estimated value of σ for each subject and the value function $\mathrm{u(M)} = -\lambda\frac{(-M)^{1-\sigma}}{1-\sigma}$ for losses (M < 0) and $\mathrm{u(M)} = \frac{M^{1-\sigma}}{1-\sigma}$ for gains (M > 0), the range of the loss aversion parameter λ for each switching point is estimated by equating the

expected utilities in columns A and B.

Risk preference experiment 2 was undertaken to measure the subjects' degree of loss aversion. The experiment involved gains and losses. Subjects were asked to choose between columns A and B; both columns were sub-divided into two, with each column representing a payoff and probability of 50% chance of winning or losing. For the sub-divided columns, column 1 involved gains while column 2 involved losses. When risk preference experiment 2 was chosen to be played for real cash, this scenario implied that out of the four balls inserted in the bingo machine to determine which game was to be played for real cash, ball 2 came out first. Then, when eight balls were inserted to determine which row would be played for real, and ball 2, for example, came out first, row 2-2 would be played for real cash. For subjects who had chosen option A in game 2, if either ball 1 or 2 came out of the bingo machine first, the subjects would receive Ushs 4,000, while if the first balls out were either balls 3 or 4, the subjects would lose Ushs 500. However, for subjects who had chosen option B, if either ball 1 or 2 came out of the bingo machine first, they would receive Ushs 6,000, while if the first balls out were either balls 3 or 4, the subjects would make a loss of Ushs 4,000. Given that all subjects were to receive a participation fee of Ushs 5,000, in the event of a loss by the subject, the money would be deducted from this fee. The experimental design was structured in such a way that even if a subject suffered a loss in risk game 2, he/she would still earn an amount from his/her participation fee. The minimum amount a subject would earn, in the event that he/she made the largest loss in risk preference experiment 2 is Ushs 1,000 (Ushs 5,000–4,000).

Figure A1: Risk Preference Experiment 1 Answer Sheet

RISK GAME 1

	A	B		Do you prefer A or B?
	① ②③④	①	②③④	
1–1	4,000	4,000	2,000	
1–2	4,000	7,000	2,000	
1–3	4,000	10,000	2,000	
1–4	4,000	13,000	2,000	
1–5	4,000	16,000	2,000	
1–6	4,000	16,000	3,000	
1–7	4,000	16,000	3,500	
1–8	4,000	16,000	4,000	

Figure A2: Risk Preference Experiment 2 Answer Sheet

RISK GAME2

	A		B		Do you prefer A or B?
	① ②	② ④	①②	③ ④	
2–1	6,000	-500	6,000	-4,000	
2–2	4,000	-500	6,000	-4,000	
2–3	1,000	-500	6,000	-4,000	
2–4	500	-500	6,000	-4,000	
2–5	500	-500	6,000	-3,000	
2–6	500	-1,000	6,000	-3,000	
2–7	500	-1,000	6,000	-2,000	
2–8	500	-1,000	6,000	-1,000	
