# Supplementary material for: Farmers’ risk preferences and rice production: Experimental and panel data evidence from Uganda
Source: PLoS One. 2019 Jul 8;14(7):e0219202. doi: 10.1371/journal.pone.0219202 (PMC6613747; doi:10.1371/journal.pone.0219202)
Supplement: S1 Table — (PDF) [file pone.0219202.s002.pdf]

S1 Table. Attrition (Probit model)

Characteristics in 2009	=1 if households were attrited
Years of experience on rice cultivation	-0.002*
	[0.001]
Age of household head	0.000
	[0.001]
Education of household head	0.008
	[0.010]
Education of household head (squared)	-0.001
	[0.001]
Number of household members	-0.006
	[0.004]
Share of male adults aged 15-64	-0.012
	[0.100]
Share of female adults aged 15-64	-0.123
	[0.095]
Land owned (ha)	-0.008
	[0.007]
Share of lowland owned	0.050
	[0.039]
Member of local group	-0.063***
	[0.020]
Owned bull	-0.009
	[0.025]
LC1 FE	Yes
Observations	599

Robust standard errors in brackets, clustered at LC1, marginal effects are shown

*** p<0.01, ** p<0.05, * p<0.1
