# Supplementary material for: Farmers’ risk preferences and rice production: Experimental and panel data evidence from Uganda
Source: PLoS One. 2019 Jul 8;14(7):e0219202. doi: 10.1371/journal.pone.0219202 (PMC6613747; doi:10.1371/journal.pone.0219202)
Supplement: S2 Table — (PDF) [file pone.0219202.s003.pdf]

S2 Table. Correlates of Risk Preferences

	Risk Aversion			Loss Aversion		
	(1)	(2)	(3)	(4)	(5)	(6)
Rice experience (years)	-0.009			-0.006		
	(0.009)			(0.008)		
Never grown rice (dummy)		0.108			0.156	
		(0.176)			(0.179)	
Married	0.0336	0.0570	0.0535	-0.397*	-0.366*	-0.407*
	(0.166)	(0.164)	(0.165)	(0.212)	(0.214)	(0.213)
Age	0.0014	0.0008	0.0014	-0.0014	-0.0020	-0.0028
	(0.0051)	(0.0053)	(0.0053)	(0.0051)	(0.0052)	(0.0053)
Head	-0.197	-0.169	-0.171	0.203	0.217	0.186
	(0.184)	(0.188)	(0.185)	(0.209)	(0.210)	(0.213)
Male	-0.0464	-0.0766	-0.0785	-0.136	-0.147	-0.126
	(0.194)	(0.197)	(0.199)	(0.218)	(0.222)	(0.220)
Schooling	0.0198	0.0213	0.0228	-0.0174	-0.0160	-0.0190
	(0.0219)	(0.0223)	(0.0228)	(0.0229)	(0.0230)	(0.0234)
Household size	-0.00924	-0.0108	-0.0120	0.0172	0.0176	0.0134
	(0.0153)	(0.0152)	(0.0156)	(0.0166)	(0.0165)	(0.0160)
Share of males (15-69)	0.212	0.258	0.241	0.219	0.228	0.169
	(0.360)	(0.362)	(0.359)	(0.375)	(0.363)	(0.373)
Share of females (15-69)	0.356	0.312	0.326	0.501	0.456	0.452
	(0.437)	(0.439)	(0.448)	(0.463)	(0.459)	(0.477)
Landholding in acre (log)	-0.0109	-0.0119	-0.0109	-0.0498	-0.0507	-0.0493
	(0.0327)	(0.0322)	(0.0333)	(0.0414)	(0.0414)	(0.0407)
Value of assets (log)	0.0231	0.0241	0.0226	0.0169	0.0177	0.0143
	(0.0519)	(0.0516)	(0.0517)	(0.0451)	(0.0443)	(0.0454)
Drought			-0.0413			0.106
			(0.167)			(0.140)
Floods			-0.178			0.533
			(0.589)			(0.411)
LC1 fixed effects	Yes	Yes	Yes	Yes	Yes	Yes
Observations	1,040	1,041	1,041	1,004	1,005	1,005
R-Squared	0.194	0.193	0.192	0.170	0.170	0.172

Numbers in parentheses are robust standard errors clustered at LC1. Estimated by OLS. ***, **, and * indicate significance at 1, 5, and 10%, respectively. Attrition weights are used.
