# Supplementary material for: Farmers’ risk preferences and rice production: Experimental and panel data evidence from Uganda
Source: PLoS One. 2019 Jul 8;14(7):e0219202. doi: 10.1371/journal.pone.0219202 (PMC6613747; doi:10.1371/journal.pone.0219202)
Supplement: S3 Table — (PDF) [file pone.0219202.s004.pdf]

S3 Table. Determinants of Rice Cultivation (Cross section data)

	Rice grown in 2009		Rice grown in 2016	
	(1)	(2)	(3)	(4)
Risk aversion	-0.020	-0.023	0.003	0.002
	(-1.068)	(-1.216)	(0.172)	(0.097)
Loss aversion	-0.043**	-0.044**	-0.034*	-0.036*
	(-2.411)	(-2.469)	(-1.815)	(-1.916)
Head Age	-0.008***	-0.008***	-0.003	-0.003
	(-4.107)	(-4.034)	(-1.473)	(-1.488)
Head Schooling	-0.018**	-0.016*	-0.001	-0.001
	(-2.087)	(-1.909)	(-0.141)	(-0.076)
Female Head	-0.121	-0.121	-0.078	-0.076
	(-1.263)	(-1.271)	(-0.709)	(-0.705)
Household Size (log)	0.026***	0.026***	0.020*	0.022*
	(2.932)	(2.895)	(1.704)	(1.925)
Share of males (15-69)	0.241	0.237	0.103	0.115
	(1.214)	(1.203)	(0.597)	(0.668)
Share of females	0.066	0.066	0.002	0.031
(15-69)	(0.317)	(0.316)	(0.007)	(0.142)
Landholding in acre	-0.015	-0.012	0.068*	0.070**
(log)	(-0.938)	(-0.744)	(1.921)	(1.966)
Value of assets (log)	0.040**	0.038**	0.023	0.022
	(2.069)	(1.988)	(0.768)	(0.737)
Non labor income	0.070	0.080	0.001	0.027
	(1.316)	(1.546)	(0.010)	(0.338)
Off farm employment	-0.032	-0.032	0.011	0.022
	(-0.527)	(-0.522)	(0.166)	(0.339)
No mobile phone	-0.039	-0.039	0.092	0.094
	(-0.673)	(-0.679)	(0.958)	(0.979)
Farmer group member	0.109*		0.065	
	(1.688)		(0.938)	
Saving group member	-0.008		0.057	
(ROSCA or VSLA)	(-0.077)		(0.861)	
LC1 fixed effects	Yes	Yes	Yes	Yes
Observations	503	503	503	503

Numbers in parentheses are robust standard errors clustered at LC1. Estimated by Probit model. Marginal effects are shown.. ***, **, and * indicate significance at 1, 5, and 10%, respectively. Attrition weights are used.
