# Supplementary material for: Farmers’ risk preferences and rice production: Experimental and panel data evidence from Uganda
Source: PLoS One. 2019 Jul 8;14(7):e0219202. doi: 10.1371/journal.pone.0219202 (PMC6613747; doi:10.1371/journal.pone.0219202)
Supplement: S4 Table — (PDF) [file pone.0219202.s005.pdf]

S4 Table. Disadoption of Rice in 2016 and Adoption in 2016

	Grew rice in both 2009 & 2016 =1 (conditional on grew rice in 2009) Probit model, dy/dx		Grew rice only in 2016=1 (conditional on not grew rice in 2009) Probit model, dy/dx	
	(1)	(2)	(3)	(4)
Risk aversion	0.010	0.008	0.027	0.036
	(0.498)	(0.406)	(0.862)	(1.119)
Loss aversion	-0.039*	-0.042**	0.009	0.003
	(-1.950)	(-2.069)	(0.324)	(0.092)
Head Age	-0.004*	-0.004	-0.002	-0.001
	(-1.678)	(-1.585)	(-0.514)	(-0.401)
Head Schooling	-0.008	-0.006	0.002	-0.001
	(-0.839)	(-0.635)	(0.170)	(-0.072)
Female Head	0.020	0.026	-0.140	-0.178
	(0.169)	(0.221)	(-0.997)	(-1.308)
Household Size (log)	0.001	0.000	0.002	0.000
	(0.062)	(0.011)	(0.101)	(0.009)
Share of males (15-69)	0.434*	0.421*	0.091	0.201
	(1.824)	(1.777)	(0.310)	(0.674)
Share of females (15-69)	0.373	0.388	-0.074	-0.129
	(1.390)	(1.440)	(-0.256)	(-0.415)
Landholding in acre (log)	-0.020	-0.014	-0.044	-0.050*
	(-1.092)	(-0.799)	(-1.625)	(-1.756)
Value of assets (log)	0.052***	0.048***	0.002	-0.007
	(3.612)	(3.305)	(0.076)	(-0.331)
Non labor income	0.040	0.060	0.069	0.085
	(0.695)	(0.987)	(0.809)	(0.956)
Off farm employment	0.029	0.034	-0.011	0.048
	(0.448)	(0.510)	(-0.112)	(0.476)
No mobile phone	-0.065	-0.073	-0.112	-0.140
	(-1.140)	(-1.265)	(-1.164)	(-1.423)
Farmer group member		-0.086		0.007
		(-1.175)		(0.060)
Saving group member		-0.111		0.423***
(ROSCA or VSLA)		(-0.883)		(3.111)
LC1 fixed effects	Yes	Yes	Yes	Yes
Mean (s.d.)	0.702	(0.458)	0.345	(0.477)
Observations	362	362	173	173

Numbers in parentheses are z-statistics. Numbers shown are marginal effects. ***, **, and * indicate significance at 1, 5, and 10%, respectively. Attrition weights are used.
