# Supplementary material for: Farmers’ risk preferences and rice production: Experimental and panel data evidence from Uganda
Source: PLoS One. 2019 Jul 8;14(7):e0219202. doi: 10.1371/journal.pone.0219202 (PMC6613747; doi:10.1371/journal.pone.0219202)
Supplement: S5 Table — (PDF) [file pone.0219202.s006.pdf]

S5 Table. Rice Cultivation by wealth category

	Rice grown in last 12 months Probit model (dy/dx)		Share of area under rice in last 12 months Tobit model (dy/dx)		Area under rice (ha) in last 12 months Tobit model (dy/dx)	
	Poorer	Richer	Poorer	Richer	Poorer	Richer
	(1)	(2)	(3)	(4)	(5)	(6)
Risk aversion	0.0456	-0.0999	-0.00635	-0.00531	-0.0144	-0.0218
	(0.0543)	(0.0795)	(0.00766)	(0.00848)	(0.0130)	(0.0208)
Loss aversion	-0.173***	-0.0742	-0.0218***	-0.00990	-0.0433***	-0.0551***
	(0.0563)	(0.0704)	(0.00769)	(0.00771)	(0.0131)	(0.0189)
Head Age	-0.0111*	-0.0166*	-0.00159*	0.000673	-0.00250*	-0.00186
	(0.00589)	(0.00856)	(0.000855)	(0.000954)	(0.00145)	(0.00235)
Head Schooling	0.00874	-0.0746**	-0.00108	-0.00930***	0.00378	-0.0109
	(0.0266)	(0.0296)	(0.00388)	(0.00313)	(0.00659)	(0.00780)
Female Head	0.361	0.0814	0.0719	-0.0310	0.0706	-0.206
	(0.434)	(0.636)	(0.0660)	(0.0762)	(0.112)	(0.192)
Household Size (log)	0.0923**	-0.0321	0.00602	-0.00722	0.0320***	0.0174
	(0.0421)	(0.0413)	(0.00625)	(0.00460)	(0.0106)	(0.0116)
Share of males	0.924	-0.458	0.132	-0.197**	0.148	-0.129
(15-69)	(0.635)	(0.818)	(0.0989)	(0.0960)	(0.168)	(0.242)
Share of females	1.033	-1.871*	-0.136	-0.174	0.0162	-0.131
(15-69)	(0.762)	(1.014)	(0.118)	(0.118)	(0.200)	(0.297)
Landholding in acre	0.0765	0.165	-0.0223*	-0.0239**	0.0541***	0.0251
(log)	(0.0750)	(0.106)	(0.0117)	(0.0112)	(0.0198)	(0.0282)
Value of assets (log)	0.0665	0.0570	0.00489	0.00577	0.000659	0.0460
	(0.0737)	(0.114)	(0.0116)	(0.0134)	(0.0197)	(0.0337)
Non labor income	-0.161	0.0664	-0.0412	0.00676	-0.0985*	0.0368
	(0.213)	(0.237)	(0.0338)	(0.0278)	(0.0575)	(0.0700)
Off farm	0.0659	-0.176	0.0400	-0.00998	0.00334	0.0686
employment	(0.239)	(0.241)	(0.0376)	(0.0281)	(0.0639)	(0.0706)
No mobile phone	0.202	0.0338	-0.0239	0.00306	-0.0232	-0.0153
	(0.260)	(0.249)	(0.0395)	(0.0290)	(0.0671)	(0.0730)
Year fixed effect	Yes	Yes	Yes	Yes	Yes	Yes
LC1 fixed effects	Yes	Yes	Yes	Yes	Yes	Yes
Observations	496	509	496	509	496	509

Numbers in parentheses are robust standard errors clustered at LC1. Marginal effects are shown.. ***, **, and * indicate significance at 1, 5, and 10%, respectively. Attrition weights are used.

Appendix Table 6: Area under rice (ha), correlated random effect tobit model (dy/dx)

	(1)	(2)	(3)	(4)
Risk aversion	-0.00580		-0.0194	
	(0.0180)		(0.0272)	
Loss aversion		-0.0691***		-0.0590**
		(0.0172)		(0.0251)
Risk aversion x Share of HH with irrigation in 2009 (LC1)			0.0161	
			(0.0716)	
Loss aversion x Share of HH with irrigation in 2009 (LC1)				-0.0303
				(0.0654)
Share of HH with irrigation in 2009 (LC1)			3.019***	2.947***
			(0.465)	(0.507)
Head Age	-0.00551***	-0.00642***	-0.00565***	-0.00609***
	(0.00208)	(0.00205)	(0.00203)	(0.00201)
Head Schooling	-0.00283	-0.00497	0.000322	-0.00292
	(0.00790)	(0.00787)	(0.00772)	(0.00772)
Female Head	-0.0211	-0.0254	-0.00626	-0.0136
	(0.157)	(0.155)	(0.157)	(0.156)
Household Size (log)	0.0323***	0.0311***	0.0329***	0.0315***
	(0.0115)	(0.0115)	(0.0116)	(0.0115)
Share of males (15-69)	0.155	0.132	0.146	0.123
	(0.214)	(0.213)	(0.214)	(0.213)
Share of females (15-69)	-0.143	-0.141	-0.171	-0.165
	(0.263)	(0.262)	(0.264)	(0.263)
Landholding in acre (log)	0.0636***	0.0675***	0.0637***	0.0672***
	(0.0239)	(0.0240)	(0.0240)	(0.0241)
Value of assets (log)	0.0361	0.0443	0.0376	0.0463*
	(0.0273)	(0.0273)	(0.0277)	(0.0276)
Non labor income	-0.0214	-0.0484	-0.0223	-0.0501
	(0.0673)	(0.0679)	(0.0676)	(0.0682)
Off farm employment	0.0276	0.0320	0.0270	0.0310
	(0.0708)	(0.0708)	(0.0711)	(0.0711)
No mobile phone	0.0406	0.0303	0.0451	0.0333
	(0.0711)	(0.0711)	(0.0714)	(0.0714)
Year fixed effect	Yes	Yes	Yes	Yes
LC1 fixed effects	Yes	Yes	Yes	Yes
Observations	1006	1006	1006	1006
