# Supplementary material for: Farmers’ risk preferences and rice production: Experimental and panel data evidence from Uganda
Source: PLoS One. 2019 Jul 8;14(7):e0219202. doi: 10.1371/journal.pone.0219202 (PMC6613747; doi:10.1371/journal.pone.0219202)
Supplement: S6 Table — (PDF) [file pone.0219202.s007.pdf]

S6 Table. Determinants of area under rice (ha)

	(1)	(2)	(3)	(4)
Risk aversion	-0.00580		-0.0194	
	(0.0180)		(0.0272)	
Loss aversion		-0.0691***		-0.0590**
		(0.0172)		(0.0251)
Risk aversion x Share of HH with irrigation in 2009 (LC1)			0.0161	
			(0.0716)	
Loss aversion x Share of HH with irrigation in 2009 (LC1)				-0.0303
				(0.0654)
Share of HH with irrigation in 2009 (LC1)			3.019***	2.947***
			(0.465)	(0.507)
Head Age	-0.00551***	-0.00642***	-0.00565***	-0.00609***
	(0.00208)	(0.00205)	(0.00203)	(0.00201)
Head Schooling	-0.00283	-0.00497	0.000322	-0.00292
	(0.00790)	(0.00787)	(0.00772)	(0.00772)
Female Head	-0.0211	-0.0254	-0.00626	-0.0136
	(0.157)	(0.155)	(0.157)	(0.156)
Household Size (log)	0.0323***	0.0311***	0.0329***	0.0315***
	(0.0115)	(0.0115)	(0.0116)	(0.0115)
Share of males (15-69)	0.155	0.132	0.146	0.123
	(0.214)	(0.213)	(0.214)	(0.213)
Share of females (15-69)	-0.143	-0.141	-0.171	-0.165
	(0.263)	(0.262)	(0.264)	(0.263)
Landholding in acre (log)	0.0636***	0.0675***	0.0637***	0.0672***
	(0.0239)	(0.0240)	(0.0240)	(0.0241)
Value of assets (log)	0.0361	0.0443	0.0376	0.0463*
	(0.0273)	(0.0273)	(0.0277)	(0.0276)
Non labor income	-0.0214	-0.0484	-0.0223	-0.0501
	(0.0673)	(0.0679)	(0.0676)	(0.0682)
Off farm employment	0.0276	0.0320	0.0270	0.0310
	(0.0708)	(0.0708)	(0.0711)	(0.0711)
No mobile phone	0.0406	0.0303	0.0451	0.0333
	(0.0711)	(0.0711)	(0.0714)	(0.0714)
Year fixed effect	Yes	Yes	Yes	Yes
LC1 fixed effects	Yes	Yes	Yes	Yes
Observations	1006	1006	1006	1006

Numbers in parentheses are robust standard errors clustered at LC1. Estimated by correlated random effect tobit model and the average marginal effects are shown. ***, **, and * indicate significance at 1, 5, and 10%, respectively. Attrition weights are used.
